# Personality Disorders Revisited: A Newly Proposed Mental Illness

**DOI:** 10.7759/cureus.9634

**Published:** 2020-08-09

**Authors:** Glen Ramsay, Ayodeji Jolayemi

**Affiliations:** 1 Psychiatry, Interfaith Medical Center, Brooklyn, USA

**Keywords:** pd, dependent personality disorder, adjustment disorder, altruistic personality disorder, altruistic depressive type, depression, personality disorders

## Abstract

Personality disorders such as dependent personality disorder (DPD), among others, have shown limited academic development in terms of a more in-depth understanding or subtypes that may exist as a mental illness or associated condition. DPD was first published as a distinct personality disorder in the Diagnostic and Statistical Manual for Mental Disorders, 3rd edition (DSM III) psychiatry manual in 1980. Since its revision in the DSM IIIR in 1987, no significant advancements have been proposed to date. This case study reported a patient with suicidal ideation and offered a new type of DPD to advance personality disorders research. The new subtype of dependent personality disorder has a few key characteristics of the traditional disorder yet reveals features that mirror nearly opposite symptom criteria, making it unique as a distinct subtype or possibly a separate personality disorder of its kind. The case study patient report proposes comorbid diagnoses of adjustment disorder and dependent personality disorder, the altruistic depressive type. Recommendations for further research were made.

## Introduction

Personality disorders such as dependent personality disorder (DPD), have shown limited academic development in terms of its contribution as a condition associated with mental illness. DPD was first published as a distinct personality disorder in the Diagnostic and Statistical Manual for Mental Disorders, 3rd edition (DSM III) psychiatry manual in 1980. Since its revision in the DSM IIIR in 1987, no significant advancements have been proposed to date [1]. The diagnosis of personality disorder presents a few challenges when seeking to diagnose mental illness-related conditions precisely. These challenges often occur because of overlapping clinical symptoms that appear in one or more personality disorder conditions. The problem is that overlapping symptoms can lead to misdiagnosis or a patient undiagnosed of his/her mental illness [2,3]. An example of overlapping-associated conditions would be an extended mental illness such as major depressive disorder (MDD). MDD has many subtypes and comorbid conditions. As a result, a thorough and careful patient and clinical history should be obtained to diagnose and identify comorbid personality disorders accurately. In many instances, when both clinical and psychiatry histories are analyzed for comorbidities, specified diagnoses may be achieved instead of a generalized unspecified diagnosis [4]. Consider the example of a patient who suppresses most of her history and only shares the latest episode of childhood sexual trauma leading to a diagnosis of post-traumatic stress disorder (PTSD) with a differential diagnosis of adjustment disorder. Precisely gathering a complete patient history including clinical symptoms of intermittent daytime shortness of breath, heart palpitations to diagnose PTSD would reduce future associated risks of suicide attempt, especially if symptoms of depression with anxiety are identified and treated early. Conversely, patients with a stressful event history diagnosed with adjustment disorder or situational depression are unlikely to develop PTSD. They are yet equally or more likely to develop suicide ideation and commit suicide. Due to the significance of harmful outcomes related to psychiatric assessment and planning, personality disorders like dependent personality disorder have resurfaced as a topic suggesting further research and development. Suicide remains a prevalent cause of death in the United States, and the connection to adjustment disorder has been systematically reviewed and found to be significant [5-7].

In the case study presented herein, Ms. L, a 71-year-old woman with a past psychiatric history of recurrent major depression and suicidal ideation, was admitted as an inpatient, later diagnosed with comorbid diagnoses of adjustment disorder and dependent personality disorder, the altruistic depressive type.

## Case presentation

Patient description

Ms. L., a 71-year-old woman with a past psychiatric history of over twenty-seven recurrent hospitalizations for major depression and suicidal ideation, walked into the emergency department herself, stating she needed help and had thoughts to jump in front of a moving car. Among all prior suicidal ideations, having intent and a plan were reported, but attempts were self-intercepted. The patient had an appropriate appearance, made good eye contact, yet spoke in a slow, low tone, with circumferential speech. She reported a depressed mood and had a congruent flat affect, profusely tearing during the interview. Suicidal ideation was expressed along with feelings and thoughts of worthlessness, after being laid off from her job as a nursing home health aide three months prior, during the COVID-19 pandemic. “I want to kill myself; I want to be with my mother, father, and brothers,” she exclaimed. The patient exhibited thought blocking and, when speaking, expressed an overabundance of thought content filled with hopelessness. She endorsed auditory hallucinations that instructed her to commit numerous self-harm methods, which included jumping in front of a car, jumping out of a window, banging her head on a desk, cutting her wrist, and overdosing on medications. The patient had limited insight into her mental illness, stating, "I'm' here because I am afraid I'm going to kill myself. I don't want to die, but I'm tired and don't want to be here anymore." She expressed good judgment and was aware of the consequences of her actions, stating, "I'm afraid to leave the hospital because If I do, I know what I will do to myself; my brother and my godson would be hurt if I took my life.”

Further evaluation and follow-up interviews revealed a deeper cause of the patient's mental illness. The patient's source of worthlessness was triggered by the loss of employment and connected to the belief that she had no one to care for, stating, "I loved taking care of my patients as a health aide. I took care of and raised all my brothers, nieces, and nephews, and they're all grown now." In fact, according to the patient, and three historian sources of collateral information gathered, the patient had never lived alone for any significant period. At the age of 10, the patient's mother suddenly separated from her father, then she, along with her eight siblings, moved away. While her mother worked, the patient, as the eldest child, helped raise her siblings. At the age of 20, the patient lived alone for five months, then subsequently had lived and continued to live with various family members for the last 51 years, until the time of this case study. There was a cyclical pattern of living with a family member to help clean, cook, or raise their children as her own. Whenever the patient did not have someone to care for, a downward spiral of emotional breakdown with thoughts of worthlessness would ensue. Suicidal ideation then followed, triggered by two significant stressors from her past: The patient witnessed her father die in her presence at the hospital due to myocardial infarction complications. At age 15, the patient was forced to have a traumatic abortion using a metal hanger during the gestational age of 7 months. "My mother took away my chance to have a child of my own. I could never have kids!” the patient exclaimed.

During the five-week treatment period in the psychiatry unit, the patient demonstrated cyclical behaviors of attachment to peer patients with individual attention. For one peer patient, she would help make a young woman’s bed daily; on another, she often provided parental-like advice. On another peer with limited mobility, she accompanied him all day long with a conversation. Each time, there was a departure of a peer, with whom the patient was attached to help, she became depressed, tearfully returning to suicidal ideation, expressing, “all my friends are leaving me here alone.” After a new attachment peer relationship formed with an existing or new peer patient admitted to the ward, these feelings subsided. Immediately after being notified of her discharge date, the patient spent time daily calling the parents of a 10-year-old child she embraced as her godson, although she has not seen him in several years.

Physical examination results

At the time of physical examination, the patient reported no signs of mental distress and reported a past medical history of right knee osteoarthritis and annual vaginal bleeding that annually occurred for one day without any complications. Vital signs and physical examination were insignificant. The patient endorsed previously using dentures that were not in her possession, loose front teeth, and no apparent dental caries.

Results of pathological tests and other investigations

Complete blood count (CBC), comprehensive metabolic panel (CMP), thyroid-stimulating hormone (TSH), diagnostic tests were ordered that resulted in no significant findings.

Personality disorder assessment and results

Administration of a personality questionnaire to assess pathological personality traits was performed utilizing the DSM-V personality assessment (The Personality Inventory for DSM-5 also known as PID-5 [8]). The personality test was performed to assess the patient's personality disorder and identify possible characteristics that align with the personality disorder identified in this case study. PID-5 has been commonly used as a reliable instrument used to identify personality disorders. Personality pathologies seen in psychiatric patients may or may not be identifiable in the DSM-V psychiatry manual.

Results from the DSV personality assessment were analyzed and categorized based on the average score derived from answers provided from the following characteristics related to dependent personality disorder, adjustment disorder, and major depressive disorder (Table 1).

**Table 1:** DSM-V Personality Assessment (PID-5) Test Results Data DSM-V is the Diagnostic and Statistical Manual for Mental Disorders, 5th edition [8].

CHARACTERISTIC	AVERAGE SCORE	PERSONALITY ASSESSMENT RESULTS
Anhedonia	(1.2 average score)	based on answer results: 3,1,1,1,1,2,0,2,1,0
Anxiousness	(2.2 average score)	based on answer results: 3,3,2,3,3,0,2,2,2
Attention seeking	(0.4 average score)	based on answer results: 0,0,0,2,0,1,0,0,0
Depressivity	(1.9 average score)	based on answer results: 2,2,2,3,2,3,2,3,0,2,2,2,0,1,3
Impulsivity	(1.3 average score)	based on answer results: 2,2,2,0,1,1
Intimacy Avoidance	(2.7 average score)	based on answer results: 2,3,3,3,3,2
Separation Insecurity	(2.0 average score)	based on answer results: 0,3,0,2,3,3,3
Withdrawal	(1.9 average score)	based on answer results: 1,1,0,2,3,2,2,3,3,2
Detachment	(1.9 average score)	based on the averaged results: Withdrawal (1.9), Anhedonia (1.2), Intimacy Avoidance (2.7)

The patient had some difficulty answering the questions. Answer selections on the personality inventory assessment ranged from very false or often false, sometimes or somewhat false, sometimes or somewhat true, very true or often true [8]. However, the patient had difficulty differentiating between the terms ‘sometimes or somewhat false’ and ‘sometimes or somewhat true.’ She often struggled with the terms ‘false or true’ and reported answering at times with a mindset of ‘sometimes less and sometimes more’ to help her answer the question prompts with greater clarification.

The personality assessment results were interpreted based on a low to high scale (0 to 3) on the form used to gauge the severity level of the personality disorder being tested. Significant findings were a low attention-seeking personality score (0.4), which aligned with the patient's independent or ‘non-dependent on others’ personality characteristics. An additional prompt was read by the patient: "I feel most happy when others depend on me." Upon reading the statement and repeating it to herself aloud, the patient responded, "Yes, that's often me" and designated a corresponding number ‘3’ to the statement.

Other interesting results findings were an anhedonia score (1.2) and a depressivity score (1.9). These scores corresponded with mild, and mild to moderate levels of depression, clinically aligned with the case study patient. During the assessment, the patient shared she was nervous about leaving and wished to remain in the hospital among the company of remaining friendships made as an inpatient instead of rejoining her family. She also gathered contact numbers from peer patients with plans to visit them after being discharged. Anxiousness moderate level results (2.2) were also consistent with the patient’s mood. The moderate to high score of intimacy avoidance (2.7) was suggestive and confirmed by the patient as infrequent desire to have sexual relations and the avoidance of intimate and long-lasting relationships due to relationship distrust seen with her parents as reported, “I had a rough childhood. I was molested as a child by my father’s friends. I saw my mother’s infidelity with another man before she left my father and took me, and my siblings then moved away."

Lastly, scores of moderate separation insecurity (2.0) and mild impulsivity (1.3), suggested that although the patient endorsed a desire for being needed for security when around others, the attachment with others wasn't a random act. It was a consistent, subconscious act of attaching to those who appear helpless or have a desired need. Evidence consistent with these results was seen numerous times during the inpatient stay at the hospital. The patient initiated no communication with roommates who appeared self-sufficient, independent, or resourceful.

In sum, the litany of traumatic psychosocial experiences endorsed by the case study patient appears to be associated with the creation of unconscious defense mechanisms. These defense mechanisms appear to be employed by psychiatric patients (not exclusive) seeking a means to manage their stressors, fears, and emotions from a traumatic history. The case study herein aspires to reignite the study of personality disorders to emphasize the importance of utilizing one’s clinical experience and art of identifying personality disorder characteristics, in efforts to achieve an early diagnosis. Establishing an initial treatment plan serves to help avoid the possibility of delayed diagnoses of primary or life-threatening psychiatric outcomes.

Treatment plan

The initial treatment plan was to stabilize patient mood with medications, provide individual cognitive therapy with coping mechanism strategy instruction, and encourage group therapy. The patient was placed on 1:1 observation monitoring to assess suicide risk. Administer the DSM V personality test for further assessment and confirm the discharge or final diagnosis.

Expected outcome of the treatment plan

The expected outcome of the treatment plan was to reach stabilization of the patient's fluctuating depressed or anxious mood with medication management. Additional outcome plans were to deeply assess collateral information from the patient and several sources to find stressors, external or medical/medication-induced influences on depressive mood, and suicidal ideation.

Actual outcome

Collateral information from several family members, various relatives, and friends that the patient had resided with over the last 51 years confirmed suspicions of comorbidity to the diagnosed mental illness of major depression induced by acute psychosocial stressors (recent unemployment). Ms. L. developed a coping mechanism that caused her to be dependent on the desire to care for others in order to boost her self-worth or mood, and her desire to continue living. Diagnosis of the patient’s personality disorder was made after five weeks of consistent and repetitive behaviors observed, reported, and described herein consistent with a disorder referred to as Dependent Personality Disorder, Altruistic Depressive Type. The altruistic depressive type specifier was added in efforts to propose the described personality disorder herein, as a limited form of dependent personality yet still, a distinct type characterized by the need to do good for others.

Treatment involved antidepressant/antianxiety medications, individual therapy, and observation of the patient providing self-therapy by helping peer patients in the unit. Ms. L. became also known in the psychiatric ward as “the lady patient who helped everyone.”

The patient was stabilized and frequently redirected with numerous relapses of transient depressive mood. Suicidal ideation occurred on a weekly and biweekly basis when peers who formed a close relationship with her, were discharged. The patient was discharged after she made arrangements to be picked up by a family friend, who agreed to have her godson join them for the summer.

## Discussion

Personality disorders are known to develop or manifest in patients as a comorbidity to other forms of mental illness [9,10]. Identifying personality characteristics associated with severe life-threatening forms such as major depression contributes to early psychiatric treatment and suicide-related mental illness prevention. When identified, personality disorder characteristics also serve to better manage suicide risks based on severity levels and support the advancement of clinical psychiatry with precise patient diagnosis.

The characteristics of the patient in the presented case study are based on 61 years of collateral history, obtained patient history and observed/documented behaviors during the inpatient period of five weeks. Clinical symptoms converted a previously diagnosed patient with a major depressive disorder to a new diagnosis of persistent or chronic adjustment disorder. However, this diagnosis offered limited specificity to the nature of the patient’s mental illness. Adjustment disorder, also known as situational depression, presented with characteristics seen in this patient, such as tearfulness, and feelings of hopelessness. The diagnosis of adjustment disorder, according to DSM V [10], is one or both of the following:

1. Marked distress out of proportion, considering external or cultural influential factors.

2. Significant impairment of social, occupational, or other functioning subtypes such as depressed mood, anxiety, mixed anxiety, depression, and mixed or conduct disturbance.

The case study patient exhibited symptoms of marked distress, which occurred when the patient was triggered by recurring stressors and thoughts of being alone. The distress incurred accompanied a depressed mood. However, it was transient and quickly relieved with positive redirection. The transient depressive mood characterized in the case study patient, although similar, is distinct from prolonged sadness seen with major depressive disorder. What makes the presented case unique is how unconscious or deep the patient’s mental illness was rooted. The patient had been so driven or obsessed with the need to be valued by helping others that it became an influential factor that led the patient to reside with other people, nearly her entire adult life. The patient was self-sufficient and highly functioning in adult daily living activities and had worked all her adult life as a health care aide. She was involuntarily laid off from work at the age of 71 years due to the Covid-19 pandemic. Yet, throughout her life, the patient chose to move from home to home, positioning herself as a cleaner, babysitter, or some form of financial help at a home, where someone was dependent and utilized her help. When the patient's family members expressed that they were no longer in need of her help, the patient rendered herself homeless and presented herself to psychiatry emergency department with suicidal ideation, no reason to live, and no place to live, despite the fact the patient was financially able to support herself. In addition, Ms. L. had numerous siblings who confirmed they were willing to have her reside with them. It was interesting to note that the patient often felt taken advantage of when family and friends over-utilized her frequent ‘acts of kindness.’ Although the patient’s mental illness presented as a form of dependence, it clinically manifested itself in a way that mirrors the opposite of the traditional dependent personality disorder. The described dependent personality disorder showed itself in an altruistic way that may even be coined as ‘depend on me personality disorder.’

The diagnosis of dependent personality disorder was updated in the DSM-IV and currently defined as a pervasive and extensive need to be taken care of and is diagnosed based on meeting 5/8 of the following criteria according to DSM V:

1. Unable to make everyday decisions without reassurance from others

2. Allows others to make important decisions in his/her life

3. Agrees with people even if they are thought to be wrong, fears the loss of approval

4. Difficulty initiating projects due to lack of self-confidence

5. Performs unpleasant and excessive tasks to obtain approval from others

6. Dislikes being alone with feelings of helplessness

7. Devastated when close relationships end and urgently seeks a replacement

8. Preoccupation with fears of abandonment and being left to care for one’s self

This case study presents a patient with only three out of eight DPD criteria suggesting a distinct personality disorder or an unspecified type of dependent personality disorder (Dependent disorder symptoms were manifested as 6. disliking being alone, 7. depressed and devastated when close relationships end, and 8. preoccupation with fears of abandonment).

Chronic adjustment disorder symptoms seen in Ms. L. manifested as excessive and consistently recurrent emotional distress on account of past psychological traumas. The distress did not present with daytime flashbacks or nightmares, feelings or symptoms of significant anxiety, nor panic. The patient’s past psychiatric history of excessive tearing and endorsement of “feeling depressed” with suicidal ideation could explain why the diagnosis of major depression was previously made on the patient and reoccurred as a diagnosis over the last 50 years, resulting in over 27 hospital admissions. It is interesting to note that despite management with medications for anti-depression treatment, the patient remained cyclically refractive, regarding her affective mood. On two occasions, when the patient initially agreed on a discharge date, on the morning of discharge, she stated, “I’m not ready. I’m afraid I’m going to hurt myself if I leave. I’m making plans to see my godson, Pooka Pooka”. Cognitive behavioral therapy and supportive psychotherapy services were referred to follow up as an outpatient telehealth treatment plan for the patient’s mental illness.

Upon discharge, the patient held a baseline mental status of good mood with congruent affect stabilized by the need to be needed, needing someone to care for, or needing someone to help. When these needs were met, the patient’s symptoms of cyclical depressive mood and suicidal ideation with hospitalization were resolved with support from various family members or friends whom she provided domestic, social, and financial support. The patient described in this case study exhibits a limited form of dependent personality disorder, yet a more specific and unique type not previously described: Dependent Personality Disorder- Altruistic, Depressive mood Type or Altruistic Depressive Disorder.

Summary of existing literature

A review of existing literature on personality disorders provided limited data. Disney published a critical analysis of dependent personality disorder (DPD), providing a detailed history of its development, to its near demise just before the American Psychiatry Association board of trustees approved the latest publication in the DSM-V psychiatry professional manual. It was interesting to note that the first time DPD was published as a distinguishable personality disorder was in 1980 in the DSM-III. Dependent personality disorder was then revised several years later in DSM-III-R. Since then, no significant additions or advancements were made with personality disorders [1]. Bachrach et al. performed a literature review with statistical data analyses and concluded that DPD is a theoretical principle with two dimensions: mental incompetence and another involving dysfunctional attachment [2].

The findings illustrated in this case study highlight the distinction of the traditional type of DPD and the newly proposed type, which has a multidimensional construct that includes dysfunctional attachment yet with functional, competent individuals with limited insight into their mental illness. The newly proposed disorder described in this case report may also be described or classified as an unconscious disorder [8-10].

Characteristics of the newly proposed disorder, DPD-Altruistic Depressive Type (DPD-ADT) or Altruistic Personality Disorder, are also found in the ‘Big Five’ Personality Traits, described as characteristics that relate to differences in behavior:

1. Openness to experience intellectual curiosity and cognitive imagination

2. Conscientiousness (organization, productiveness, responsibility)

3. Extraversion (social ability, assertiveness)

4. Agreeableness (compassion, respectfulness, trust in others)

5. Neuroticism (tendencies toward anxiety and depression)

DPD-ADT character qualities found in the patient case study possesses all the Big 5 personality traits. Traditional DPD was reported via meta-analysis to correlate with only two of the five personality qualities, Agreeableness and Neuroticism. Other studies also reported qualities of low enthusiasm and extraversion of DPD yet documented opposite findings consistent with DPD-ADT. High levels of excitement and extroversion were also correlated with Altruistic Personality Disorder [9, 11-14].

Risk factors for developing DPD have been studied and reported to be associated with social anxiety disorder (SAD). Children who develop SAD have a 10-time increased risk of developing dependent personality disorder [15].

Another personality disorder with overlapping character qualities with abandonment fear is borderline personality disorder (Borderline PD). Both DPD and Borderline PD are strongly associated with dependency [7]. Literature review revealed other studies that supported overlapping symptom criteria by reporting 30-40% of patients with borderline personality disorder also have a comorbid diagnosis of DPD [9]. A good prognosis for DPD was published in the DSM IV, stating that the real or perceived return of the caregiver’s nurturance results in the remission of symptoms [4]. The similarities between borderline PD and Dependent PD are increased tendencies for dysfunctional attachment to others or forms or dependence and increased risks of suicidality. The distinction between the two personality disorders is the presence of relationship violence or psychosis, which is a more common characteristic with borderline PD [16-20].

The etiology for DPD is supported by the notion that family upbringing, social-observed learning, and childhood trauma have been implicated as a causative role in dependent personality disorder. The case study patient’s history aligns with childhood trauma as a causative role outlined in reported literature [1, 4, 12]. It makes logical sense that children who were separated from their caregivers in some way may become adults with developed attachment or detachment issues connected to mental illness. Alexander described supportive findings in a study and concluded that children receive lessons about themselves and the world through experiences with their caregivers, which serve as a model later to be emulated positively or negatively. A child or an adult's negative attributes are evidenced by viewing one’s self as dysfunctional or inadequate and acquiring insecure attachment relationships with others [4].

It is not surprising that cognitive behavioral therapy is a treatment method for DPD (Dependent PD) of various subtypes. Cognitive therapy enables a patient diagnosed with a personality disorder to understand his/her history, including childhood traumas. A patient's treatment of relearning how to relate with his or her past facilitates the transformation of thinking from feeling inadequate to mentally strong men and women who view themselves as functionally adequate when alone. The self-sufficient capacity to fill themselves up, without a need to depend on others or have feelings of emptiness resolved by helping others, indicates successful treatment [4].

To date, the altruistic type of dependent personality disorder has not been previously found on literature search engines as a topic extensively reviewed. Contribution of another specific type of dependent personality disorder linked to adjustment disorder, among others, will contribute to the psychiatry field of study and will serve clinicians who seek to exercise mastery over making a precise diagnosis and enhancing the treatment of mental illness disorders. It is recommended that more studies are done on personality disorders to utilize them as identifiable markers or primary diagnosis indicators of mental illness. A skilled clinician's capacity to efficiently conduct a psychiatry interview with personality disorders in mind may increase the likelihood of achieving a prompt clinical diagnosis. Further advancement of dependent personality disorder (DPD) subtypes and associative conditions such as adjustment disorder can spark a renaissance toward the advancement of clinical psychiatry.

## Conclusions

Dependent Personality Disorder, Altruistic Depressive Type, when approved for the next latest edition of DSM-VI, will add an enhanced level of diagnosing affective mood disorders with higher specificity. The proposed diagnostic criteria below may also need ICD-11 diagnostic coding for clinician selection as a specific sub-type diagnosis.

Dependent personality disorder, altruistic depressive type (also referred to as altruistic personality disorder) or adjustment disorder, altruistic depressive type: A mental condition where patients have a depressive mood when separated from the act of helping others. The need to help others fills a void or emptiness within. They have an excessive need to help others or have others dependent on them. When detached from the self-initiated dependent relationship, fear of loneliness and excessive depressive mood occurs that quickly resolves when replaced by another attachment or dependency relationship.

Symptoms of dependent personality disorder, altruistic depressive type (also referred to as altruistic personality disorder): A-L-T-R-U-M-I-S-T-I-C

Adjustment mood disorder association

Loves attaching to others who need help or provide feelings of validation, and worthiness

Terrified of abandonment or have thoughts of self-harm when unable to attach to others

Relationships are self-initiated and created for dependency on him/her

Unconscious or unpredictable behavior

Major depression look-alike yet presents as a transient depressive mood

Inability to be alone; immediately replaces one relationship with another

Suicidal ideation

Transforms into the ‘victim’ role to gain empathy support for worthlessness

Independently can care for oneself

Cyclically and emotionally labile: sad or tearfully depressed to a happy mood
